# Differential expression of calcium-dependent protein kinase 4, tubulin tyrosine ligase, and methyltransferase by xanthurenic acid-induced *Babesia bovis* sexual stages

**DOI:** 10.1186/s13071-021-04902-3

**Published:** 2021-08-10

**Authors:** Hala E. Hussein, Wendell C. Johnson, Naomi S. Taus, Janaina Capelli-Peixoto, Carlos E. Suarez, Michelle R. Mousel, Massaro W. Ueti

**Affiliations:** 1 Department of Veterinary Microbiology and Pathology, Washington State University, Pullman, WA USA; 2 Department of Entomology, Faculty of Science, Cairo University, Giza, Egypt; 3 The US Department of Agriculture-ARS-Animal Disease Research Unit, Pullman, WA USA; 4 Paul G. Allen School for Global Animal Health, Washington State University, Pullman, WA USA

**Keywords:** *Babesia bovis*, Calcium-dependent protein kinase 4 (CDPK4), Methyltransferase, *Rhipicephalus microplus*, Xanthurenic acid

## Abstract

**Background:**

*Babesia bovis* is one of the most significant tick-transmitted pathogens of cattle worldwide. *Babesia bovis* parasites have a complex lifecycle, including development within the mammalian host and tick vector. Each life stage has developmental forms that differ in morphology and metabolism. Differentiation between these forms is highly regulated in response to changes in the parasite’s environment. Understanding the mechanisms by which *Babesia* parasites respond to environmental changes and the transmission cycle through the biological vector is critically important for developing bovine babesiosis control strategies.

**Results:**

In this study, we induced *B*. *bovis* sexual stages in vitro using xanthurenic acid and documented changes in morphology and gene expression. In vitro induced *B*. *bovis* sexual stages displayed distinctive protrusive structures and surface ruffles. We also demonstrated the upregulation of *B*. *bovis* calcium-dependent protein kinase 4 (*cdpk4*), tubulin-tyrosine ligase (*ttl*), and methyltransferase (*mt*) genes by in vitro induced sexual stages and during parasite development within tick midguts.

**Conclusions:**

Similar to other apicomplexan parasites, it is likely that *B*. *bovis* upregulated genes play a vital role in sexual reproduction and parasite transmission. Herein, we document the upregulation of *cdpk4*, *ttl*, and *mt* genes by both *B*. *bovis *in vitro induced sexual stages and parasites developing in the tick vector. Understanding the parasite's biology and identifying target genes essential for sexual reproduction will enable the production of non-transmissible live vaccines to control bovine babesiosis.

**Graphical abstract:**

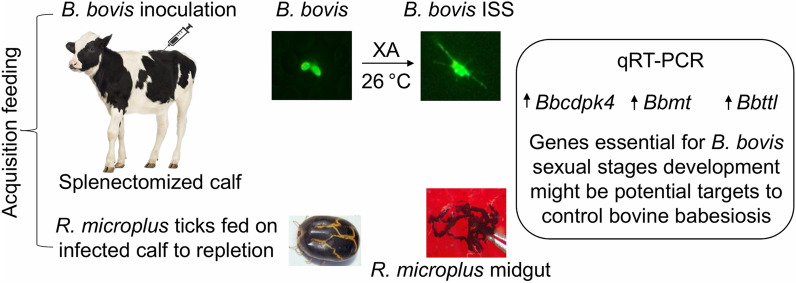

**Supplementary Information:**

The online version contains supplementary material available at 10.1186/s13071-021-04902-3.

## Background

Bovine babesiosis caused by *Babesia bovis* is one of the most important tick-borne diseases of cattle in tropical and subtropical regions [1]. *Babesia bovis* is transmitted by *Rhipicephalus* larval ticks [2, 3]. As the infected larval ticks feed on the vertebrate host, *B*. *bovis* sporozoites are inoculated via saliva into the host’s bloodstream [2], where they invade and replicate asexually in red blood cells (RBC) as merozoites [4, 5]. When *R*. *microplus* ticks feed on infected animals, they ingest *B*. *bovis*-infected erythrocytes. In the midgut of the tick, *Babesia* undergoes sexual reproduction, which develops into the kinete stage that circulates in tick hemolymph. After kinete invasion of eggs, the parasites are transmitted transovarially [6]. This results in larval progeny containing *B*. *bovis* sporozoites that can infect cattle [2].

The clinical signs of bovine babesiosis are characterized by high fever, hemolytic anemia, anorexia, inappetence, hemoglobinuria, and capillary parasite sequestration, leading to death when adult cattle are acutely infected [7]. However, young animals are more resistant to acute infections than adult cattle (> 1 year old). Younger animals’ innate immune system plays an important role in controlling the disease, especially through the clearance of infected erythrocytes in the spleen [6]. Parasites can also establish persistent infections in the absence of obvious clinical signs. These infections lead to inapparent and chronic infections. However, tick transmission of the parasite to other hosts can occur [6].

*Babesia bovis* transmission relies on the production of sexual stages by responding to environmental signals inside the tick midgut lumen [8]. Disruption of *B*. *bovis* development in the tick midgut would prevent transmission via tick vectors. To date, there are no methods to isolate sexual stages from infected tick midgut. We artificially induced *B*. *bovis* sexual stage development in in vitro cultures using xanthurenic acid (XA), an intermediate metabolite derived from tryptophan metabolism [9, 10]. In vitro induction of *B*. *bovis* sexual stages allowed identifying sexual stage-specific genes and gene families such as the *hap2* gene, the cystine motif-rich gene family, and the *ccp* gene family [10–12]. These genes encode proteins that may be important candidates for developing an effective transmission-blocking vaccine to control bovine babesiosis [10–12].

In this study, we examined the differential gene expression of *B*. *bovis* calcium-dependent protein kinase 4 (*cdpk4*), tubulin-tyrosine ligase (*ttl*), and methyltransferase (*mt*) upon XA induction. *Babesia bovis* CDPK4, TTL, and MT proteins are homologs to previously identified gamete-specific proteins in *Plasmodium falciparum* [13] and *B*. *bigemina* [14]. We also describe the effect of XA on parasite morphology associated with the development of sexual stages. The present study aimed to understand the basic biology of *Babesia* parasites and to identify additional sexual stage genes that will enable the development and production of novel *Babesia* vaccines and possible drug targets to control bovine babesiosis.

## Methods

### Cattle, ticks, and parasite cultures

A splenectomized 3–4 months male Holstein that tested negative for *B*. *bovis* by PCR [2] and cELISA [15] was used in this study. The animal was maintained according to protocols approved by the University of Idaho Institutional Animal Care and Use Committee (IACUC #2018-16). *Rhipicephalus microplus* La Minita tick strain [16] was used. Approximately 40,000 larvae from 2 g of eggs were placed under a cloth patch to feed. When approximately 1% of the nymphs molted to adults, the calf was inoculated intravenously with *B*. *bovis* S_74_T_3_Bo strain stabilate containing approximately 1 × 10^7^ *B*. *bovis*-infected erythrocytes [17] to synchronize the peak of parasitemia with female tick repletion. The infected calf was monitored daily for *B*. *bovis* in peripheral blood and clinical signs of babesiosis [18] (Additional file 1: Fig. S1). Replete female ticks were collected, washed in tap water, dried, and incubated at 26 °C with 93% relative humidity. During the development of *B*. *bovis* within the tick midgut, five engorged ticks were removed daily from the incubator and dissected for 6 consecutive days. Individual midgut was placed into 1 ml of TRIzol^®^ reagent (Thermo Fisher Scientific, Waltham, MA) and stored at − 80 °C. Eleven days post-*B*. *bovis* inoculation, infected defibrinated blood was collected from the calf, and the erythrocytes were washed five times with Puck’s saline G to remove white blood cells. Washed infected RBCs were pelleted by centrifugation at 3000 rpm, 10 min at 4 °C, and suspended in TRIzol.

### Induction of in vitro* B*. *bovis* sexual stages

To induce sexual stages, *B*. *bovis* infected blood was collected and maintained in in vitro cultures for 1 week before induction. The in vitro cultured *B*. *bovis* infected erythrocytes were suspended in a medium with or without 100 μM XA (Sigma, St. Louis, MO, USA) as previously described [10]. Induced in vitro sexual stage parasites were isolated at 12 h and 24 h post-induction by differential centrifugation at 400×*g* for 1 min. The supernatant was recovered and the sexual stages pelleted at 2000×*g* for 5 min. The sexual stage parasites were suspended in TRIzol and stored at − 20 °C. To estimate cell viability, cells were suspended in PBS, mixed with an equal volume of 20 μg/ml 6-carboxyfluorescein diacetate [19] in PBS (Calbiochem-Behring, La Jolla, CA, USA), and incubated at room temperature for 15 min. The cells were then washed twice with PBS and visualized by a Leica microscope using LAS-X software.

### Scanning electron microscopy

Induced sexual stages were washed three times with PBS. Samples were fixed in 2% paraformaldehyde, 2% glutaraldehyde, and 0.1 M phosphate buffer and incubated overnight at 4 °C. Samples were rinsed twice with distilled water and then post-fixed overnight in 2% osmium tetroxide. After rinsing, parasites were dehydrated with an ethanol series (30–100%). Final drying was with hexamethyldisilazane (HMDS). Approximately 50–100 µl of sample suspended in HMDS was pipetted onto a coverslip attached to an aluminum SEM stub and placed in a vacuum desiccator overnight [20] before gold coating. Samples were imaged on a FEI SEM Quanta 200F at Franceschi Microscopy and Imaging Center, Washington State University.

### Transmission electron microscopy

Sexual stage parasites from induced cultures were washed three times with PBS. Samples were fixed in 2% paraformaldehyde, 2% glutaraldehyde, and 0.1 M phosphate buffer and refrigerated overnight at 4 °C. Fixed samples were microwaved in a Pelco Biowave Pro 36500 Laboratory Microwave System for 2 min at 750 Watts, with a temperature cutoff restriction set at 28 °C. Samples were rinsed twice with distilled water and post-fixed overnight in 1% osmium tetroxide at 4 °C. After rinsing, the parasites were dehydrated with an ethanol series (30–100%), then placed into propylene oxide (PO) and infiltrated overnight in a 1:1 PO:Spurr resin mix. Infiltration medium was poured off and changed to 100% Spurr’s embedding media overnight and samples polymerized at 65 °C before thin sectioning (80–100 nm) [21]. Sections were stained sequentially with uranyl acetate, potassium permanganate, and Reynold’s lead before observation with an FEI Tecnai G2 TEM.

### In silico target gene identification by genomic search and bioinformatic analysis

To identify *B*. *bovis* homologs of *P*. *falciparum* and *B*. *bigemina* gamete-specific proteins CDPK4, MT, and TTL, bioinformatic analysis was performed based on amino acid identity using NCBI Blastp (https://blast.ncbi.nlm.nih.gov/Blast) and the complete annotated *B*. *bovis* genome sequence (https://www.ncbi.nlm.nih.gov/nuccore/AAXT00000000.2) [22]. Clustal Omega analysis (http://www.ebi.ac.uk/Tools/msa/clustalo/) was used to evaluate the percent amino acid identity of proteins. Protein domains conserved among *B*. *bovis*, *P*. *falciparum*, and *B*. *bigemina* homologs were determined using the Simple Modular Architecture Research Tool (http://smart.embl-heidelberg.de/). Transmembrane domains were predicted for the target proteins using the Transmembrane Hidden Markov Model Package 2 (TMHMM2) [23] (http://www.cbs.dtu.dk/services/TMHMM-2.0). SignalP-5.0 was used to predict putative signal peptides [24]. Multiple alignments of CDPK4 amino acid sequences from *B*. *bovis*, *B*. *bigemina*, *T*. *equi*, *T*. *parva*, and *P*. *falciparum* were generated using Multiple Sequence Alignment by CLUSTALW (http://www.genome.jp/tools/clustalw/). Cello v2.5 predictor was used to predict protein translocation and subcellular localization [25] (http://cello.life.nctu.edu.tw/). Phylogenetic analysis of CDPK4 from *B*. *bovis*, *B*. *bigemina*, *T*. *equi*, *T*. *parva*, and *P*. *falciparum* was conducted using Phylogeny.fr.software; the tree prediction is based on an approximate likelihood-ratio test (aLRT) as an alternative to nonparametric bootstrap and Bayesian estimation of branch support [26, 27] that used *MUSCLE* for alignment, *Gblocks* for curation, *PhyML* for phylogeny, and *TreeDyn* for graphic representation.

### RNA extraction and cDNA synthesis

Total RNA was extracted from blood, induced sexual stages, and tick gut samples using TRIzol reagent according to manufacturer’s protocol and the RNA pellets suspended in 20 µl DEPC-treated water. RNA samples were treated with DNase I (Invitrogen, Waltham, MA, USA) following the manufacturer’s protocol to remove contaminating genomic DNA, quantified by Nanodrop (Thermo Fisher Scientific, Waltham, MA, USA). The removal of genomic DNA was confirmed by PCR targeting rap1 as previously described [28] using non-reverse transcribed samples. cDNA was synthesized from 150 ng total RNA of each sample with the Superscript^®^ First-strand cDNA synthesis kit (Invitrogen, Waltham, MA, USA) following the manufacturer’s protocol.

### Quantitative PCR assay and primer design

To examine the expression pattern of *B*. *bovis cdpk4*, *mt*, *ttl*, and *hap2*, specific primers for each gene were designed using the PrimerQuest^®^ Tool (Integrated DNA Technologies (IDT)) (Table 1) following recommended guidelines for qPCR primer design. Primers were purchased from Eurofins Genomics (Louisville, KY, USA). BLASTn analysis confirmed that primer sequences were not contained in the *R*. *microplus* genome (NC_023335.1). Melting curve analysis was added to the PCR cycle to check the specificity of each primer pair. Standard PCR was performed to amplify the full-length gene from cDNA samples for all target genes using primers listed in Table 1. PCR cycling conditions consisted of 95 °C for 3 min followed by 35 cycles of 95 °C for 30 s, 55 °C for 30 s, and 72 °C for 2 min, with a final extension of 72 °C for 5 min. PCR products were visualized by 1% agarose gel electrophoresis. PCR amplicons were cloned into PCR 2.1-TOPO^®^ (Thermo Fisher Scientific) and submitted for sequencing (Eurofins MWG Operon, Louisville, KY). Standard curves were generated for each gene using specific quantities of plasmids. For the normalization of the qPCR data, *B*. *bovis α-tubulin* and *mitogen-activated protein kinase* (*mapk*) genes were evaluated as parasite reference gene candidates. CFX Manager™ software (Bio-Rad, Hercules, CA, USA) [29] was used to examine the stability of expression of the reference gene candidates. The qPCRs for the genes of interest and reference gene candidates were performed in a CFX96™ Real-Time PCR Detection System (C1000 Touch™ Thermal Cycler) (Bio-Rad, Hercules, CA, USA) using the SsoFast™ EvaGreen^®^ Supermix Kit (Bio-Rad, Hercules, CA, USA). The cycling conditions consisted of initial denaturation at 95 °C for 2 min followed by 40 cycles of 95 °C denaturation for 15 s and annealing at 55 °C for 30 s. Reactions were performed in triplicate in 20 μl using 300 nM of each primer and 2 μl of 1/20 dilution of cDNA as template. qPCR for *msa-1* was performed to amplify a 150-bp fragment between bases 604 and 754 of *msa-1* (GenBank Accession number AF275911) using *msa-1*-specific primers, fluorogenic probe, and SsoAdvanced Universal Probes Supermix (Bio-Rad, Hercules, CA, USA) as previously described [2]. A standard curve was developed using dilutions of specific numbers of *msa-1* plasmid as previously described [2]. CFX Manager™ software (Bio-Rad, Hercules, CA, USA) was used to analyze the qPCR data. Amplification efficiency was evaluated to determine the sensitivity of the qPCR for each gene. Relative expression was calculated with a division of each gene detected expression by *mapk* detected expression within each time point. There were heterogeneous variances between time points, and therefore relative expression was transformed to log_10_. Differences due to time were tested with a mixed linear model with a fixed effect of time and repeated effect of technical replicate. Pairwise differences of time were tested with Tukey or Tukey-Kramer (unbalanced data) adjustment.

**Table 1:** Gene identification and primer sets of *Babesia bovis* genes of interest used for PCR and quantitative PCR

Gene identification^a^	Locus tag	Forward primers (5′-3′)	Reverse primers (5′-3′)	Size^b^
*cdpk4* (FL)	BBOV_IV003210	CGCTGCTAAAGTGCAACATATCTTT	CGTGTATGCATTTAGACACCTAGTTT	1707
*cdpk4* (qPCR)	BBOV_IV003210	GGCAGTATGTCGGACAAGGT	CGAACGATCCTTTACCCAGA	193
*mt* (FL)	BBOV_II003780	ATGACAGAACTTGCCCATGATCTC	GAAACAGTCTGTAACCTGCGTTT	234
*mt* (qPCR)	BBOV_II003780	TGACAGAACTTGCCCATGATC	GGGGAAACATCTTCTTCATCTCA	102
*ttl* (FL)	BBOV_III004540	ATGTTAAAGTCAGACATACCCATG	TCTTTGAAAAATTGCAAGTGG	1242
*ttl* (qPCR)	BBOV_III004540	TACACTGGGAATTGCACGAA	GACGCCGTGGGTACTTTTTA	205
*mapk* (FL)	BBOV_IV005520	CTCCATTGTACAAGTGCCCAAAGGAG	CATGGCTTGTATATAATTTTGAGTGG	2203
*mapk* (qPCR)	BBOV_IV005520	GCTTACGTAACCCGCCACTA	ATATCAAAGGCACGGCAGAC	151
*α-tubulin* (FL)	BBOV_III002820	GCCAACTTCAATCACTTCATTCCG	GATGCTACGATTAAGTAAATGTTTTTC	1463
*α-tubulin* (qPCR)	BBOV_III002820	CATGCTTGACAACGAGGCTA	TGCGAGGGTAAGGTACCAAG	188
*hap2* (qPCR)	BBOV_III006770	AAAGCGTCTATGTAATCAA	ACAGTTTTCTTCTCGTCA	165

*FL* full length, *qPCR* quantitative PCR

^a^Information in parentheses refers to the purpose of PCR. Full-length primers used to amplify constructs used to build qRT-PCR standard curves

^b^Amplicon size in base pairs

## Results

### Induction and morphological analysis of in vitro* B*. *bovis* sexual stages

**Fig. 1:**
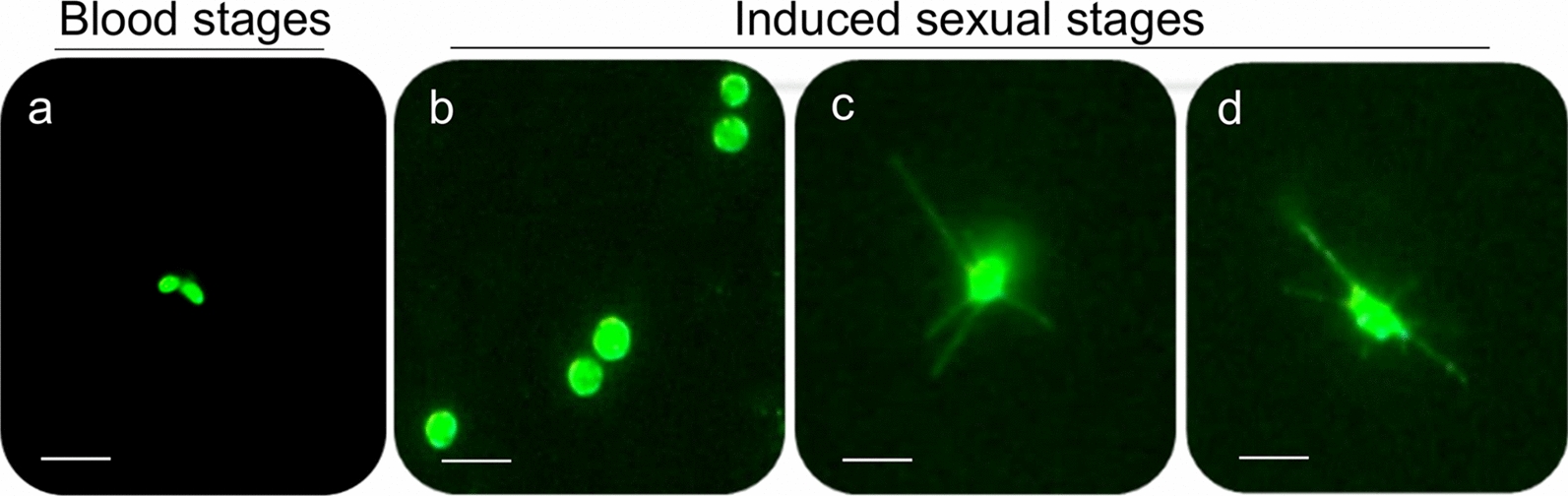
Morphology of *B*. *bovis* blood stages and induced sexual stages stained with 6-CFDA. **a** Blood stages. **b**–**d** Sexual stages induced in in vitro culture using XA at 26 °C. Scale bar: 5 µm

**Fig. 2:**
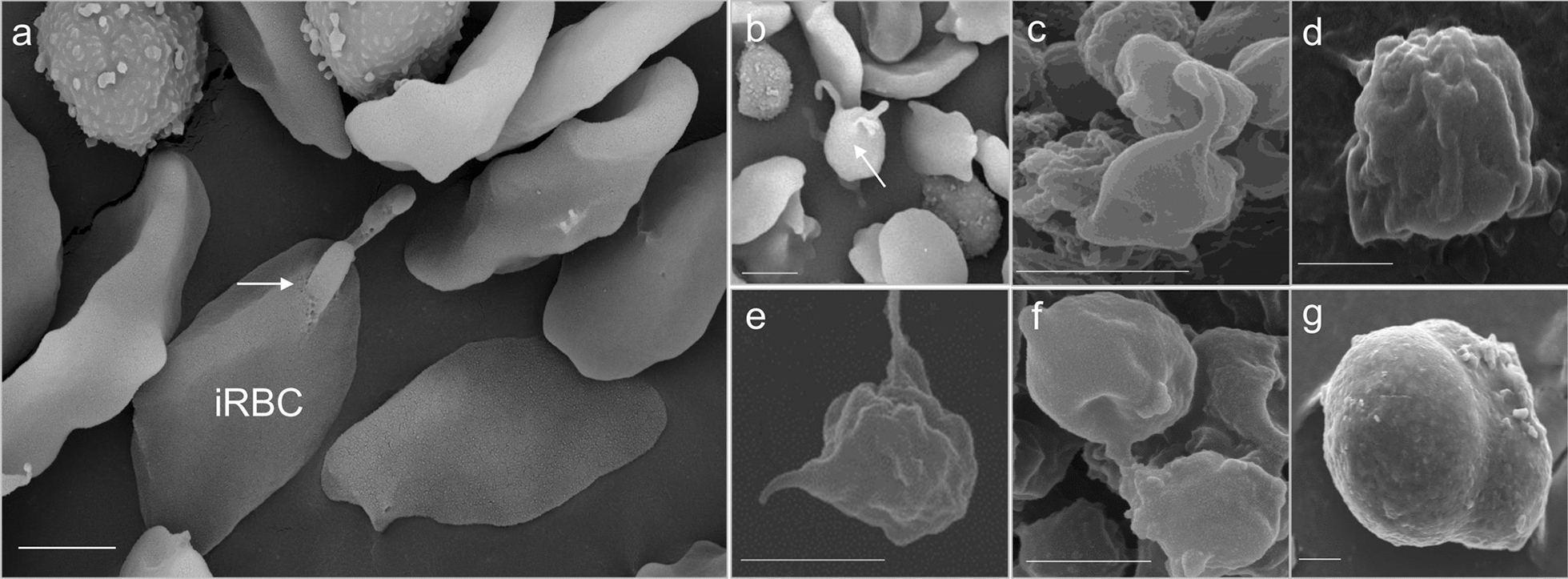
Scanning electron micrographs of induced in vitro* B*. *bovis* sexual stages. **a** Lysis of the infected red blood cell (iRBC) membrane and release of *B*. *bovis* sexual stages (white arrow) from erythrocyte observed 3 h post-induction. **b** Extracellular *B*. *bovis* induced parasite (black arrow) observed 12 h post-induction, **c**–**e** Exflagellation of *B*. *bovis* sexual stages observed 12 h post-induction. **f**, **g** Parasite fusion 24 h post-induction. Scale bar: 2 µm

**Fig. 3:**
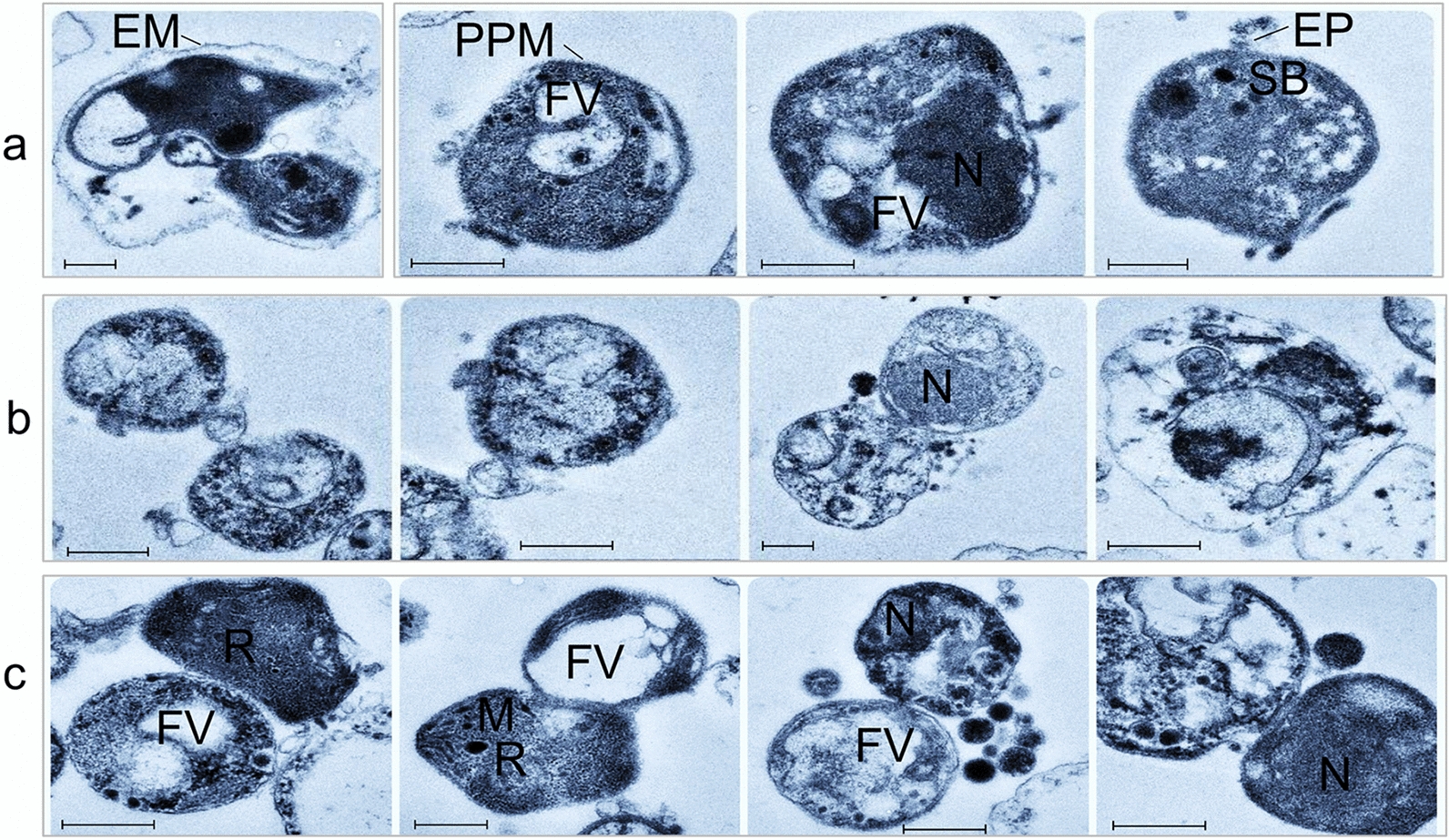
Transmission electron micrograph of ultrastructural changes in *B*. *bovis* upon XA sexual stage induction. The sections were made at time points **a** 3 h, **b** 12 h, and **c** 24 h after addition of XA. *N* nucleus, *FV* food vacuole, *EP* external processes, *PPM* parasite plasma membrane, *M* microneme, *R* rhoptry, *EM* erythrocyte membrane. The polymerized samples were 80–100 nm thin sections. Scale bar: 500 nm

Microscopic inspection of non-induced *B*. *bovis* blood stages showed pyriform-shaped parasites in RBC (Fig. 1a). In contrast, *B*. *bovis* cultures induced by decreasing temperature to 26 °C and addition of xanthurenic acid to the culture media showed the presence of extra-erythrocytic parasites with long projections and large round parasite stages, indicative of parasite sexual stage formation (Fig. 1b–d). Analysis of XA-induced cultured parasites using scanning electron microscopy showed that egress of in vitro-induced sexual stage parasites from infected RBC began by 3 h after the onset of induction (Fig. 2a). *Babesia bovis* induced sexual stage cells at 12 h post-XA induction displayed elaborate and distinctive protrusive structures, such as projections and surface ruffles (Fig. 2b–e). *Babesia bovis* induced sexual stages were found to undergo cell-to-cell fusion, forming multinucleated syncytia, upon XA in vitro induction (Fig. 2f, g). Ultrastructural analysis of induced parasites using transmission electron microscopy showed parasites were mostly rounded or slightly ovoid, exhibiting distinct, unique features that are characteristic of in vitro induced *B*. *bovis* sexual stages. Typical *Babesia* organelles, such as prominent double-membrane nuclei, mitochondria, apicoplasts, spherical bodies, rhoptries and micronemes, vacuoles, and numerous free ribosomes, were observed in extracellular *B*. *bovis* sexual stages. Some parasites contained abundant cytoplasmic organelles, while others presented large vacuoles in the cytoplasm. Sequential ultrastructural studies of *B*. *bovis* sexual stages showed a pattern of coordinated development of sexual forms, starting with parasite egress from host RBC concomitant with the rupture of the infected RBC membrane (RBCM) observed 3 h after induction (Fig. 3a). The process culminated with parasite cell-to-cell membrane fusion and the formation of multinucleated syncytia (Fig. 3b, c). However, nuclear fusion was not observed.

### In silico analysis of target genes

In silico analysis was performed to select *B*. *bovis* homologs to previously identified sexual stage genes in *P*. *falciparum* (*cdpk4*, *mt*, and *ttl*) and *B*. *bigemina* (*mt*, *ttl*). In silico predictions suggested the absence of signal peptide and transmembrane domains in *B*. *bovis* CDPK4, MT, and TTL proteins. Also, these proteins were found to be cytosolic, consistent with their homologs in *P*. *falciparum* [13, 30]. Overall, *B*. *bovis* CDPK4, MT, and TTL amino acid sequences appeared well conserved compared with homologs in other species. Accession numbers and functional annotation of *B*. *bovis* target proteins are shown (Table 2). *Babesia bovis* CDPK4 contains a serine/threonine protein kinase, catalytic domain (STKc_CAMK), and an EF-hand type Ca (2+) binding domain (Additional file 2: Fig. S2). *Babesia bovis* CDPK4 (GenBank: XP_001609485.1) shares 60%, 85%, 77%, and 68% amino acid identity with CDPK4 from *P*. *falciparum* (GenBank: XP_001349078.1), *B*. *bigemina* (GenBank: XP_012766984.1), *T*. *equi* (GenBank: XP_004833801.1), and *T*. *parva* (GenBank: XP_766594.1), respectively. Multiple alignments and phylogenetic analysis of amino acid sequences corresponding to the CDPK4 of *B*. *bovis*, *B*. *bigemina*, *T*. *equi*, *T*. *parva*, and *P*. *falciparum* revealed conservation among apicomplexan parasites (Additional file 3: Fig. S3) consistent with the taxonomic evolution of *B*. *bovis* and related apicomplexan species (Additional file 4: Fig. S4). *Babesia bovis* MT (GenBank: XP_001609901.1) contains a conserved methyltransferase domain (Additional file 2: Fig. S2) that shares 55% amino acid identity with *B*. *bigemina* MT (GenBank: XP_012767431.1) and 33% with *P*. *falciparum* MT (GenBank: XP_001348700.2). *Babesia bovis* TTL (GenBank: XP_001611585.1) possesses a tubulin-tyrosine ligase domain (Additional file 2: Fig. S2) and shares 57% amino acid identity with *B*. *bigemina* TTL (GenBank: XP_012769325.1) and 27% amino acid identity with *P*. *falciparum* gamete-specific protein TTL (GenBank: XP_002808689.1) (Table 3).

**Table 2:** Accession numbers and functional annotation of *Babesia bovis* genes of interest

Locus_tag	Chromosome number	Accession number of *B*. *bovis* genes of interest	Accession number of *B*. *bovis* proteins of interest	Function annotation	Length cDNA (bp/aa)
BBOV_IV003210	4	XM_001609435.1	XP_001609485.1	Calcium-dependent protein kinase 4 (CDPK4)	1707/517
BBOV_II003780	2	XM_001609851.1	XP_001609901.1	Hypothetical protein (MT)	234/77
BBOV_III004540	3	XM_001611535.1	XP_001611585.1	Tubulin-tyrosine ligase family protein (TTL)	1242/413
BBOV_IV005520	4	XM_001610431.1	XP_001610481.1	Mitogen-activated protein kinase (MAPK)	2203/584
BBOV_III006770	3	XM_001611756.1	XP_001611806.1	Membrane protein (HAP2)	2274/757
BBOV_I003060	1	XM_001608906.1	XP_001608956.1	Merozoite surface antigen-1(MSA-1)	1130/ 319

**Table 3:** Protein sequence identities (%) between *Babesia bovis* homologs of *Plasmodium falciparum* and *Babesia bigemina* sexual stage-specific proteins

*B*. *bovis* protein name	*B*. *bovis* accession number	*P*. *falciparum* accession number	Identity	*B*. *bigemina* accession number	Identity	Function annotation
CDPK4 (BBOV_IV003210)	XP_001609485.1	XP_001349078.1	60	XP_012766984.1	85	Calcium-dependent protein kinase 4
MT (BBOV_II003780)	XP_001609901.1	XP_001348700.2	33	XP_012767431.1	55	Hypothetical protein (Methyltransferase)
TTL (BBOV_III004540)	XP_001611585.1	XP_002808689.1	27	XP_012769325.1	57	Tubulin-tyrosine ligase family protein

### Expression of target genes in blood stages, induced sexual stages, and tick midgut-specific stages

Quantitative PCR was used to analyze the transcription pattern of *B*. *bovis cdpk4*, *mt*, and *ttl* genes in blood from an acutely infected animal, in non-induced culture (0 h), in cultures at 26 °C without or with XA at time points 12 h and 24 h, and in tick-specific stages from individual engorged tick midgut (MG) samples collected from *B*. *bovis* infected females. *Babesia bovis α-tubulin*, *mapk* transcripts were evaluated as *B*. *bovis* reference gene candidates for data normalization (Additional file 5: Table S1). The *α-tubulin* was considered inadequate for qPCR normalization because it was not stably expressed in the tested parasite stages. In contrast, *mapk* was stably expressed throughout different time points and the development of tick-specific stages and was selected as a reference gene for normalization. Based on this, the transcription levels of all the target genes were normalized to the *mapk* expression level. The melt curve analyses showed the absence of primer dimers and nonspecific amplification for all tested genes; the efficiency of amplification ranged between 94 and 109% (Additional file 6: Fig. S5).

**Fig. 4:**
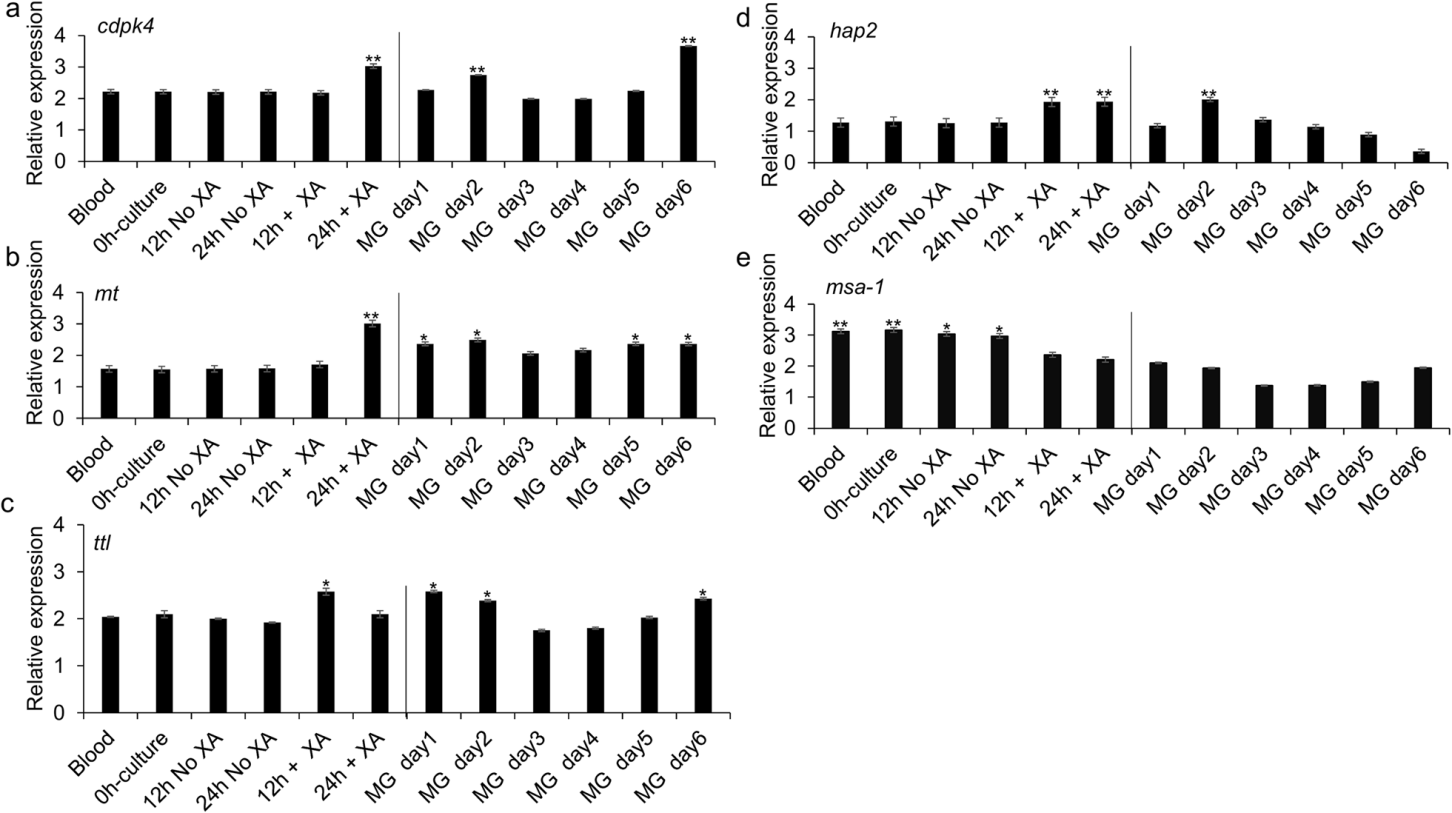
Relative expression of *B*. *bovis* genes in blood from an acutely infected animal, in non-induced culture (0 h), and cultures at 26 °C without or with XA at time points 12 h and 24 h, and tick-specific stages from individual engorged female tick midgut (MG) samples collected for 6 consecutive days after incubation (MG day1 to day 6). **a** Calcium-dependent protein kinase 4 (*cdpk4*). **b** Methyltransferase (*mt*). **c** Tubulin-tyrosine ligase (*ttl*). **d** Hapless 2 (*hap2*). **e** Merozoite surface antigen-1 (*msa-1*) genes. The data represent the mean of three experiments, each containing three technical replicates. Asterisk (*) indicates statistical pairwise differences between time points (*P* < 0.05)

The data indicate that *cdpk4* gene expression is significantly upregulated in induced sexual stages (induced by decreasing temperature to 26 °C and addition of XA to the culture media) at 24 h (*P* < 0.0001) and tick-specific stages at days 2 and 6 compared to blood stages (Fig. 4a). In addition, the level of the *mt* transcript was also increased in induced sexual stages at 24 h (*P* < 0.0234) and tick-specific stages at days 1 to 6 compared to blood stages (Fig. 4b). However, expression of *ttl* was higher in induced sexual stages at 12 h (*P* < 0.0262) and tick-specific stages at days 1, 2, and 6 compared to blood stages (Fig. 4c). Expression of the *hap2* gene was found to be upregulated in induced sexual stages at 12 h and 24 h (*P* < 0.0485) and by tick-specific stages day 2 compared to blood stages (Fig. 4d). Also, *hap2* gene expression was previously demonstrated using reverse transcription PCR (RT-PCR) to be exclusively transcribed by induced sexual stages and during *B*. *bovis* development within the tick midgut [10]. Interestingly, *msa-1* expression was found to be maximal by blood stages (*P* < 0.0019) and gradually reduced over time during sexual stage induction and tick-specific stages (Fig. 4e). The results represent the mean of three experiments, each containing three technical replicates (Additional file 7: Table S2). Taken together with the morphological analysis, these results affirmed that XA and a drop-in temperature play an important role during *B*. *bovis* sexual stage formation in the in vitro induction system used in these experiments.

## Discussion

In the present study, we examined gene expression and morphology associated with developing sexual stages in induced in vitro cultures and midguts of replete *R*. *microplus* fed on *B*. *bovis* infected calves. Herein, we extend the number of genes upregulated during *B*. *bovis* sexual stage development to include *cdpk4*, *ttl*, and *mt*. CDPK4, a serine/threonine kinase, is an enzyme that plays an important role in intracellular calcium signaling in plants, green algae, ciliates, and apicomplexan parasites [31]. In malaria parasites, CDPK4 is known to be involved in stage-specific cellular responses to calcium signaling transduction pathways, cell cycle regulation, and life cycle progression [32]. In *P*. *berghei* male gametocytes, CDPK4 is transcriptionally upregulated [30] and involved in sexual reproduction. Importantly, inhibition of *P*. *falciparum* CDPK4 blocked malaria transmission [33–35]. *Plasmodium falciparum* TTL is involved in post-translational C-terminal tyrosination of α-tubulin and regulates the formation of microtubule interacting proteins [13]. *Plasmodium falciparum* MT plays a fundamental role in gene regulation through the methylation of histones and non-histone proteins such as transcription factors which are essential for parasite development and differentiation [36]. In *B*. *bigemina*, MT was identified as a marker of tick stage development [14]. Previous studies demonstrated that differential expression of *hap2* [10], the cystine motif-rich gene family [11], and the *ccp* gene family [12] was associated with apicomplexan sexual stage development. Also, basal *B*. *bovis* kinete-specific protein (ksp) expression by blood-stage parasites was found to be upregulated by tick stage parasites [37]. However, KSP protein is restricted to tick stage parasites [37]. These observations suggest that the expression of specific proteins is required for parasite development within the tick vector. The data presented here provide evidence for the importance of *cdpk4*, *ttl*, and *mt* in the development of *B*. *bovis* sexual stages [10]. This is supported by our previous work on *B*. *bigemina* that found *ttl* and *mt* genes were upregulated in sexual stages from tick midgut or induced in vitro culture using tris 2-carboxyethyl phosphine [14]. In *P*. *falciparum*, *cdpk4*, *ttl*, and *mt* are upregulated in sexual stages and involved in sexual reproduction and arthropod infection [13, 32, 34, 36, 38]. After *B*. *bovis* sexual and zygote formation, parasites develop into kinetes in midgut epithelial cells that migrate to tick hemolymph by day 6 after repletion [37]. Upregulation of *B*. *bovis cdpk4*, *ttl*, and *mt* expression at day 6 after female tick dropping suggests that these genes may also be important in *B*. *bovis* kinete development. This agrees with a recent RNA-seq study that showed all three genes were highly upregulated in *B*. *bovis* kinetes [39].

It has been reported that XA is important for in vitro induction of *Babesia* sexual stages [8, 10, 40]. In malaria, XA is present in the gut of *Anopheles* mosquitos and is known to induce gametogenesis of *P*. *falciparum* [9]. It has recently been shown that XA supported the growth of a tick-borne pathogen, *Anaplasma phagocytophilum*, in tick cells by inhibition of tryptophan dioxygenase activity [41]. However, it remains unknown whether this metabolite is present in the tick midgut [10]. *Babesia bovis* sexual stages induced in vitro by XA were observed by light microscopy to have distinct morphological and ultrastructure features similar to those described for *B*. *canis*, *B*. *bigemina*, and *B*. *bovis* sexual stages derived from tick midgut or *Boophilus microplus* cell cultures [8, 42]. Sexual stages of *Babesia* are characterized by cytoplasmic projections and microtubule development [8, 10, 43]. Using SEM, we observed aggregation of in vitro induced parasite strahlenkörper forms similar to *B*. *bovis* and *B*. *bigemina* developing within the tick midgut [8, 44, 45]. This aggregation may improve contact between individuals before the fusion of sexual stages. The close juxtaposition of individuals was confirmed using SEM and TEM, similar to previous work on *B*. *bovis* [10].

Further experiments are necessary to investigate the role of *B*. *bovis* CDPK4, MT, and TTL proteins during parasite development within the invertebrate host. Knocking out *cdpk4*, *mt*, and *ttl* genes using gene editing and transfection techniques will facilitate determining whether disrupting these genes interferes with the parasite’s life cycle within the tick vector.

## Conclusions

Understanding the development of parasite sexual stages is considered a key component of future transmission blocking vaccines and the control of bovine babesiosis. Proteins encoded by the genes described in this study, such as CDPK4, TTL, and MT, might be potential drug or vaccine targets. The current study is a part of our ongoing research to understand *B*. *bovis* sexual stage development to design strategies to block parasite transmission.

## Supplementary Information

**Additional file 1: Fig. S1.** Clinical signs of *B*. *bovis* infection. a: Calf packed cell volume (PCV) and temperature recorded after *B*. *bovis* inoculation and during the tick feeding period. b: Giemsa-stained blood smear at day 11 after parasite inoculation showing *B*. *bovis* iRBC.

**Additional file 2: Fig. S2.** Schematic representation of the location and number of functional domains in upregulated sexual stage genes. *Babesia bovis* calcium-dependent protein kinase 4 (CDPK4) had N-terminal serine/threonine kinase domain (S_TKc) and a C-terminal calmodulin-like domain with four EF hand motifs (EF); *B*. *bovis* methyltransferase (MT) had a methyltransferase domain (MT), and *B*. *bovis* tubulin tyrosine ligase (TTL) had a tubulin-tyrosine ligase domain (TTL).

**Additional file 3: Fig. S3.** Multiple alignment of amino acid sequences corresponding to the CDPK4 of *B*. *bovis* (GenBank: XP_001609485.1), *B*. *bigemina* (GenBank: XP_012766984.1), *T*. *equi* (GenBank: XP_012766984.1), *T*. *parva* (GenBank: XP_766594.1), and *P*. *falciparum* (GenBank: XP_001349078.1). Stars correspond to a high level of conservation between species.

**Additional file 4: Fig. S4.** Phylogenetic tree based on CDPK4 amino acid sequences from *B*. *bovis* (GenBank: XP_001609485.1), *B*. *bigemina* (GenBank: XP_012766984.1), *T*. *equi* (GenBank: XP_012766984.1), *T*. *parva* (GenBank: XP_766594.1), and *P*. *falciparum* (GenBank: XP_001349078.1). CDPK4 sequence from *Chlamydomonas reinhardtii* (GenBank: XP_001693482.1) was used as outgroup for phylogenetic rooting.

**Additional file 5: Table S1.** Expression of *B*. *bovis* reference gene candidates during development of blood stages, sexual stages from in vitro induced parasites and tick midgut stages.

**Additional file 6: Fig. S5.** Standard curves of qRT-PCR assays designed for *B*. *bovis* genes. a: Calcium-dependent protein kinase 4 (*cdpk4*), b: methyltransferase (*mt*), c: tubulin-tyrosine ligase (*ttl*), d: mitogen-activated protein kinase (*mapk*), and e *hap2*. The figure shows standard curves obtained using tenfold dilutions of the construct prepared for each target gene and the reference gene *mapk* diluted from 10^7^ to 10^2^.

**Additional file 7: Table S2.** Expression of *B*. *bovis* genes essential for sexual stage development.

## Data Availability

All data generated or analyzed in this study are included within the article.

## Abbreviations

*ttl*: Tubulin tyrosine ligase
*α-tubulin*: Alpha tubulin
*cdpk4*: Calcium-dependent protein kinase 4
*mapk*: Mitogen-activated protein kinase
*mt*: Methyltransferase
*msa-1*: Merozoite surface antigen-1
*hap2*: Hapless 2
XA: Xanthurenic acid
RBC: Red blood cell
niRBC: Non-infected red blood cell
RBCM: Red blood cell membrane
ISS: Induced sexual stages
TCEP: Tris 2-carboxyethyl phosphine
bp: Base pair
PBS: Phosphate-buffered saline
PCR: Polymerase chain reaction
qPCR: Quantitative polymerase chain reaction
RT-PCR: Reverse transcriptase polymerase chain reaction
cDNA: Complementary DNA
