# Supplementary figures and images for: Differential expression of calcium-dependent protein kinase 4, tubulin tyrosine ligase, and methyltransferase by xanthurenic acid-induced Babesia bovis sexual stages
Source: Parasit Vectors. 2021 Aug 10;14:395. doi: 10.1186/s13071-021-04902-3 (PMC8353865; doi:10.1186/s13071-021-04902-3)

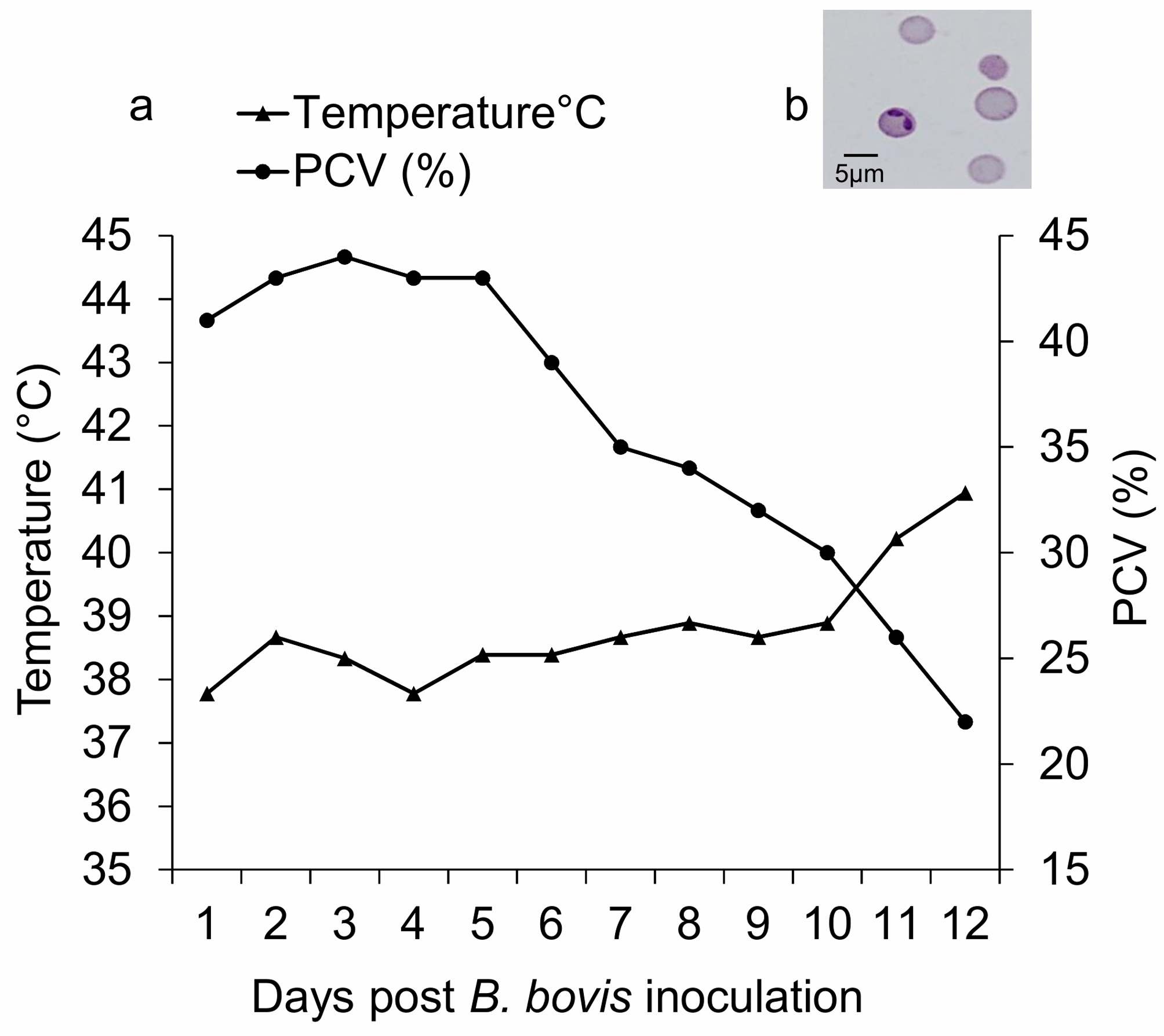

Supplement: Supplementary file 1 — Additional file 1: Fig. S1. Clinical signs of B. bovis infection. a: Calf packed cell volume (PCV) and temperature recorded after B. bovis inoculation and during the tick feeding period. b: Giemsa-stained blood smear at day 11 after parasite inoculation showing B. bovis iRBC. [file 13071_2021_4902_MOESM1_ESM.tif]

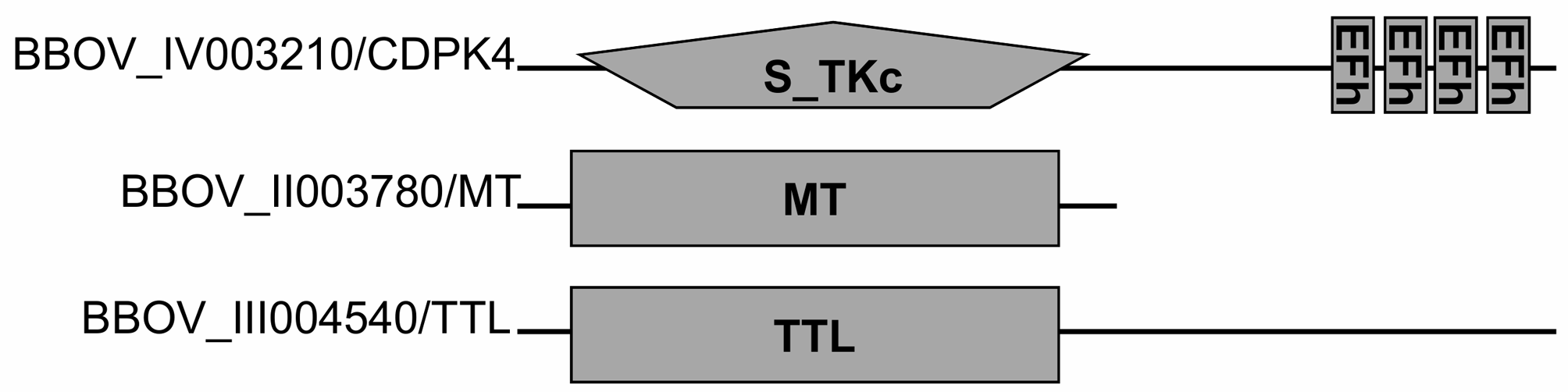

Supplement: Supplementary file 2 — Additional file 2: Fig. S2. Schematic representation of the location and number of functional domains in upregulated sexual stage genes. Babesia bovis calcium-dependent protein kinase 4 (CDPK4) had N-terminal serine/threonine kinase domain (S_TKc) and a C-terminal calmodulin-like domain with four EF hand motifs (EF); B. bovis methyltransferase (MT) had a methyltransferase domain (MT), and B. bovis tubulin tyrosine ligase (TTL) had a tubulin-tyrosine ligase domain (TTL). [file 13071_2021_4902_MOESM2_ESM.tif]

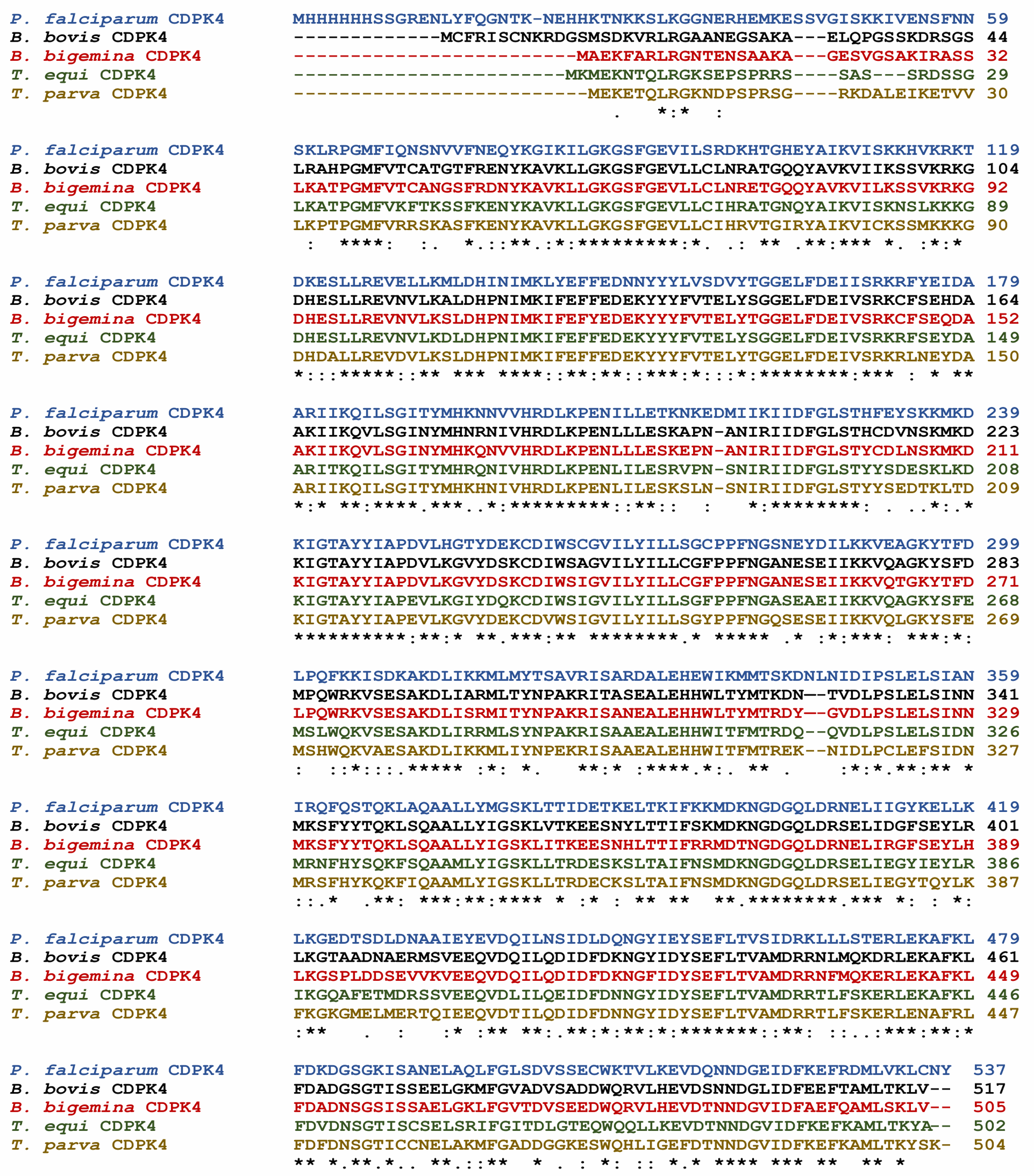

Supplement: Supplementary file 3 — Additional file 3: Fig. S3. Multiple alignment of amino acid sequences corresponding to the CDPK4 of B. bovis (GenBank: XP_001609485.1), B. bigemina (GenBank: XP_012766984.1), T. equi (GenBank: XP_012766984.1), T. parva (GenBank: XP_766594.1), and P. falciparum (GenBank: XP_001349078.1). Stars correspond to a high level of conservation between species. [file 13071_2021_4902_MOESM3_ESM.tif]

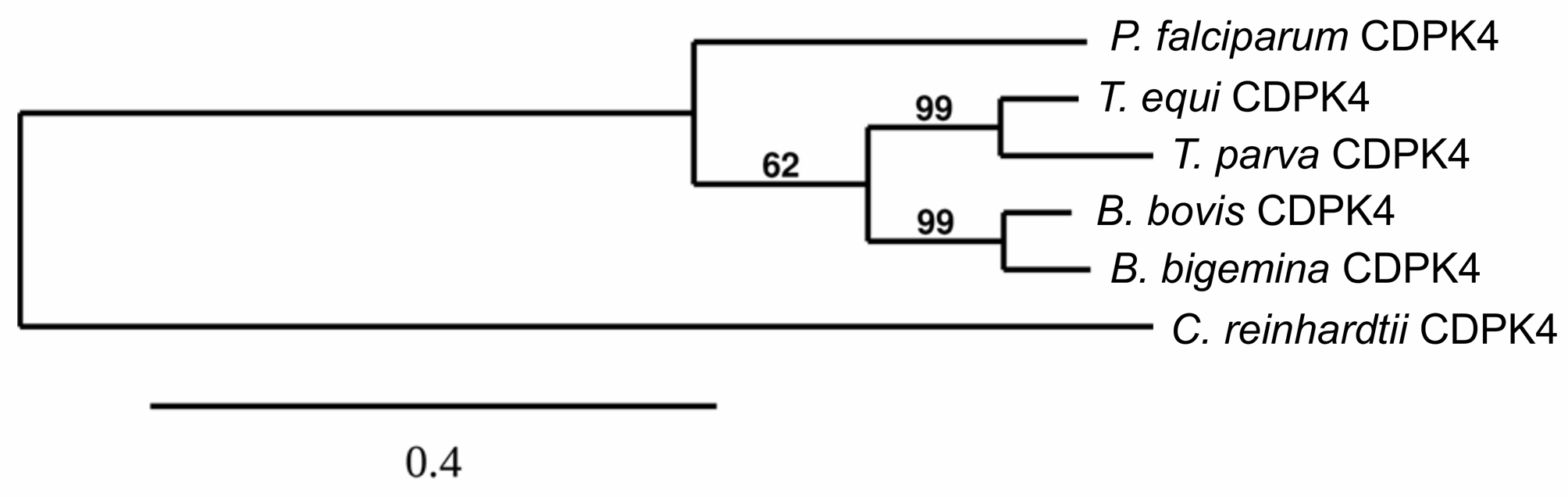

Supplement: Supplementary file 4 — Additional file 4: Fig. S4. Phylogenetic tree based on CDPK4 amino acid sequences from B. bovis (GenBank: XP_001609485.1), B. bigemina (GenBank: XP_012766984.1), T. equi (GenBank: XP_012766984.1), T. parva (GenBank: XP_766594.1), and P. falciparum (GenBank: XP_001349078.1). CDPK4 sequence from Chlamydomonas reinhardtii (GenBank: XP_001693482.1) was used as outgroup for phylogenetic rooting. [file 13071_2021_4902_MOESM4_ESM.tif]

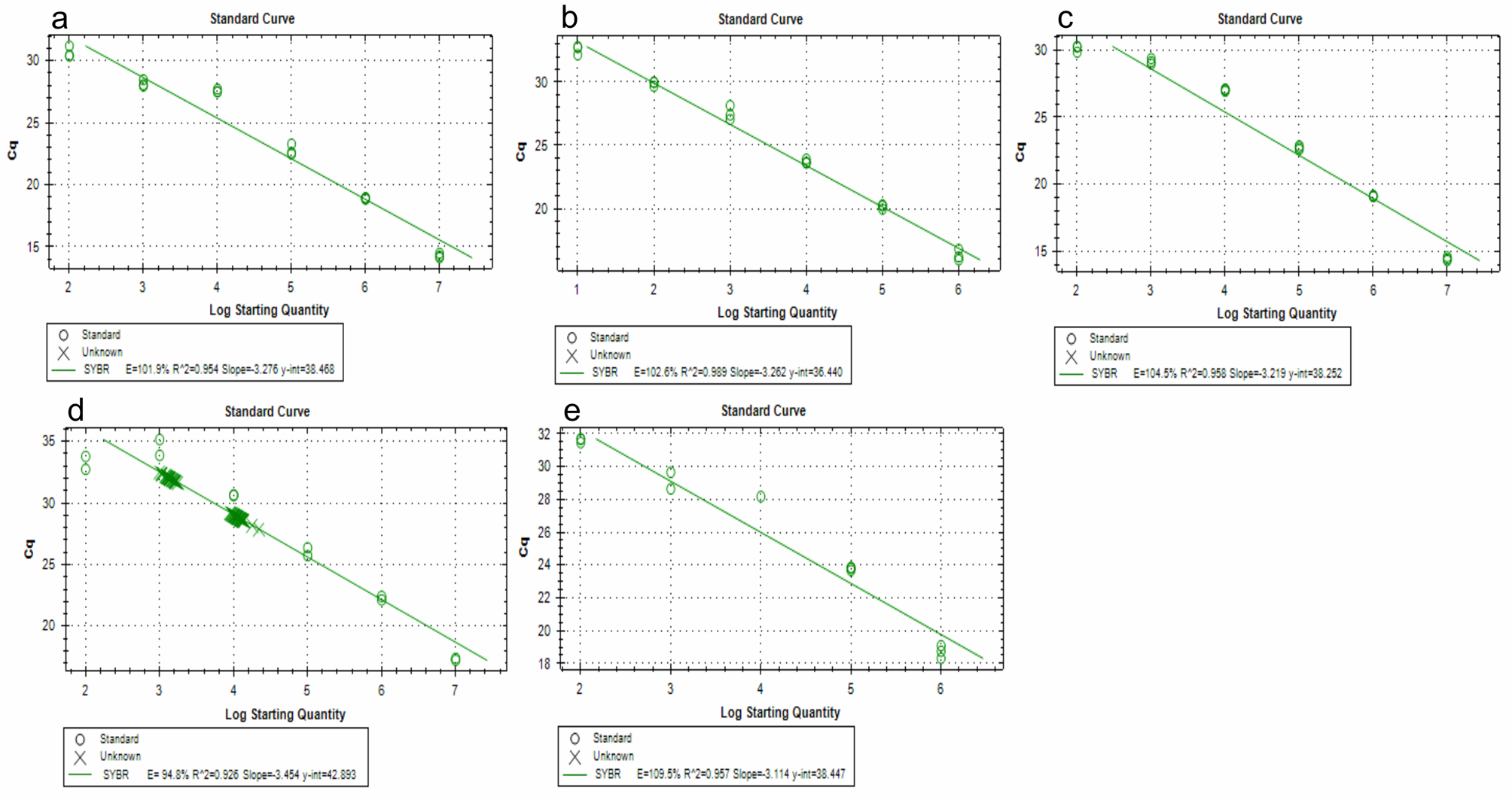

Supplement: Supplementary file 6 — Additional file 6: Fig. S5. Standard curves of qRT-PCR assays designed for B. bovis genes. a: Calcium-dependent protein kinase 4 (cdpk4), b: methyltransferase (mt), c: tubulin-tyrosine ligase (ttl), d: mitogen-activated protein kinase (mapk), and e hap2. The figure shows standard curves obtained using tenfold dilutions of the construct prepared for each target gene and the reference gene mapk diluted from 107 to 102. [file 13071_2021_4902_MOESM6_ESM.tif]
